# F130. INCREASED RISK OF PSYCHOTIC DISORDERS IN AFRICAN MIGRANTS TO AUSTRALIA

**DOI:** 10.1093/schbul/sby017.661

**Published:** 2018-04-01

**Authors:** Linglee Downey, Scott Eaton, Kristen Thien, Melissa Bardell-Williams, Meghan Bowtell, Aswin Ratheesh, Patrick D McGorry, Brian O’Donoghue

**Affiliations:** Orygen, the National Centre of Excellence in Youth Mental Health

## Abstract

**Background:**

Certain migrants groups are at an increased risk of psychosis compared to the native-born population, however these findings relate to certain countries, mainly in Europe and America where the research has been conducted. It is not yet known whether migrants to Australia are at an increased risk for developing a psychotic disorder. This study aimed to determine whether first-generation migrants in a geographically defined catchment area in Melbourne have an increased risk of developing a psychotic disorder.

**Methods:**

This study included an all young people aged between 15 and 24 residing in a geographically defined catchment area of north western Melbourne who presented to the Early Psychosis Prevention and Intervention Centre (EPPIC) between 01.01.11 and 31.12.13. Data pertaining to the at risk population was obtained from the Australian 2011 Census and incidence rates ratios were calculated.

**Results:**

A total of 527 individuals with FEP were included, 393 were Australian-born (74.6%) and 134 (25.4%) were overseas-born. First generation migrants from Kenya (IRR=9.81), Ethiopia (IRR=5.17), Somalia (IRR=3.78), and Sudan (IRR=3.57), had significantly increased risk of having a psychotic disorder. Conversely, first generation migrants from India and China had significantly decreased risk of having psychosis.

**Discussion:**

First-generation migrants from East Africa and the Horn of Africa have significantly high rates of psychosis and they may have experienced factors pre-, during, and post-migration, predisposing them to psychosis

